# Distinguishing different types of attention deficit hyperactivity disorder in children using artificial neural network with clinical intelligent test

**DOI:** 10.3389/fpsyg.2022.1067771

**Published:** 2023-01-11

**Authors:** I-Cheng Lin, Shen-Chieh Chang, Yu-Jui Huang, Terry B. J. Kuo, Hung-Wen Chiu

**Affiliations:** ^1^Graduate Institute of Biomedical Informatics, College of Medical Science and Technology, Taipei Medical University, Taipei, Taiwan; ^2^Department of Psychiatry, Shuang Ho Hospital, Taipei Medical University, New Taipei City, Taiwan; ^3^Department of Psychiatry, Taipei Municipal Wan Fang Hospital, Taipei Medical University, Taipei, Taiwan; ^4^Department of Psychiatry and Psychiatric Research Center, Taipei Medical University Hospital, Taipei, Taiwan; ^5^Institute of Brain Science, National Yang Ming Chiao Tung University, Taipei, Taiwan; ^6^Clinical Big Data Research Center, Taipei Medical University Hospital, Taipei, Taiwan; ^7^Bioinformatics Data Science Center, Wan Fang Hospital, Taipei Medical University, Taipei, Taiwan

**Keywords:** neural network, machine learning, attention deficit, hyperactivity, artificial intelligence

## Abstract

**Background:**

Attention deficit hyperactivity disorder (ADHD) is a well-studied topic in child and adolescent psychiatry. ADHD diagnosis relies on information from an assessment scale used by teachers and parents and psychological assessment by physicians; however, the assessment results can be inconsistent.

**Purpose:**

To construct models that automatically distinguish between children with predominantly inattentive-type ADHD (ADHD-I), with combined-type ADHD (ADHD-C), and without ADHD.

**Methods:**

Clinical records with age 6–17 years-old, for January 2011–September 2020 were collected from local general hospitals in northern Taiwan; the data were based on the SNAP-IV scale, the second and third editions of Conners’ Continuous Performance Test (CPT), and various intelligence tests. This study used an artificial neural network to construct the models. In addition, *k*-fold cross-validation was applied to ensure the consistency of the machine learning results.

**Results:**

We collected 328 records using CPT-3 and 239 records using CPT-2. With regard to distinguishing between ADHD-I and ADHD-C, a combination of demographic information, SNAP-IV scale results, and CPT-2 results yielded overall accuracies of 88.75 and 85.56% in the training and testing sets, respectively. The replacement of CPT-2 with CPT-3 results in this model yielded an overall accuracy of 90.46% in the training set and 89.44% in the testing set. With regard to distinguishing between ADHD-I, ADHD-C, and the absence of ADHD, a combination of demographic information, SNAP-IV scale results, and CPT-2 results yielded overall accuracies of 86.74 and 77.43% in the training and testing sets, respectively.

**Conclusion:**

This proposed model distinguished between the ADHD-I and ADHD-C groups with 85–90% accuracy, and it distinguished between the ADHD-I, ADHD-C, and control groups with 77–86% accuracy. The machine learning model helps clinicians identify patients with ADHD in a timely manner.

## 1. Introduction

Attention deficit hyperactivity disorder (ADHD) is a well-studied topic in child and adolescent psychiatry. According to the Diagnostic and Statistical Manual of Mental Disorders, Fifth Edition (DSM-5), ADHD can be divided into three subgroups, combined-type ADHD (ADHD-C), predominantly inattentive-type ADHD (ADHD-I), predominantly hyperactive/impulsive ADHD (ADHD-H; Association AP, 2013). Clinically, the most common subtypes are ADHD-I and ADHD-C (Willcutt, 2012).

In the past, the diagnosis of ADHD relied heavily on information from teachers, parents, and psychological assessment. When teachers find that students have inattention, hyperactivity and other ADHD-related behavioral problems, they may suggest that the parents take their children to the hospital for evaluation. One study showed that even in a student with strong cognitive ability, teachers were more likely to consider the possibility of ADHD because of inconsistencies between student’s behavioral problems and high cognitive levels (Degroote et al., 2022). In addition, teachers may be more aware of children with combined-type ADHD, but have less awareness about inattentive subtype of ADHD (Moldavsky et al., 2013). In clinical practice, the first diagnostic tool for patients with suspected ADHD is an assessment scale, which uses information provided by the child’s parents or teachers. The Conners’ Rating Scale (Conners, 1997) and the Swanson, Nolan, and Pelham (SNAP) scale (Swanson et al., 1981) are the most common assessment scales. The SNAP-IV scale has been translated into Chinese by Professor Gau et al. (2008, 2009). The second diagnostic tools are computer-based tests (Ballard, 1996; Forbes, 1998; Nichols and Waschbusch, 2004), the most common of which is Conners’ Continuous Performance Test (CPT; Conners, 1994). The CPT has a second edition (CPT-2; Conners, 2000) and a third edition (CPT-3; Conners, 2014), and previous studies have demonstrated the validity of both editions (Erdodi et al., 2014; Ord et al., 2020). Some cases also required the evaluation of inconsistent assessment results because the observations of teachers and parents tend to contradict each other, with an atypical cross-informant reliability of 0.32–0.59 (Power et al., 2001). Teachers’ reports using SNAP-IV scale about hyperactivity and impulsivity behavior may be more helpful for the clinical diagnosis of ADHD (Hall et al., 2020). Furthermore, the overall CPT results may only correlate with the hyperactivity score of the assessment scale (McGee et al., 2000). A previous study observed no significant correlations between the CPT Overall Index score and the parent and teacher ratings of inattentive and hyperactive behaviors (Edwards et al., 2007). Extensive clinical experience is required for psychiatrists to interpret the complex data gathered from such assessments, and psychiatrists with little exposure to children with ADHD may be overwhelmed.

Machine learning, deep learning, and artificial intelligence are widely applied technologies in medical informatics, such as in the diagnosis of dementia and Alzheimer’s disease (Thung et al., 2017; Choi et al., 2018). One study used CPT data from children with and without ADHD in a machine prediction model, which exhibited an accuracy rate of 87%, sensitivity rate of 89%, and specificity rate of 84% (Slobodin et al., 2020). A study combined clinical rating scales and psychological testing data using various assessment scales and the d2 Test of Attention to construct a decision tree and distinguish between patients with and without ADHD. Bledsoe and colleagues reported an accuracy of 100% when using Conners’ Restlessness/Impulsive Index Scale and the d2 Test of Attention and an accuracy of 97% when using the Behavioral Assessment Scale for Children, Second Edition; Hyperactivity Scale; and d2 Test of Attention (Bledsoe et al., 2016). That study only surveyed 23 patients with ADHD and 12 controls, and the results may thus generalize poorly to a larger population. Nevertheless, physicians in Taiwan use the d2 Test of Attention relatively infrequently, a study tried to develop and apply the d2-test principles to the Tien Character Attention Test (Lin and Lai, 2017). The decision tree is likely to be unsuitable for patients in Taiwan.

A notable study in Taiwan by Cheng et al. used a support vector machine method to identify ADHD based on the CPT-2, the SNAP-IV scale, and Conners’ Parent and Teacher Rating Scales-Revised: Short Form (Cheng et al., 2020), and their method achieved an accuracy of approximately 89% in experiments. They also used deep learning to impute missing values for incomplete scales. However, that study did not distinguish between ADHD-I and ADHD-C. In addition, the data did not include measurements of intelligence; some studies have reported that intelligence may affect CPT and assessment scale scores. For example, one study identified IQ to be a significant predictor of CPT-II performance (Munkvold et al., 2014). Another study indicated that certain cognitive functions exhibited a weak negative correlation with CPT results and that intelligence exhibited weak negative correlations with the cognition and inattention items in Conners’ Teacher Rating Scale (Naglieri et al., 2005). Intelligence could also affect the clinical manifestation of ADHD.

Therefore, from previous studies, we found that machine learning can help distinguish ADHD and control groups, but less attempts have been made to distinguish ADHD-I and ADHD-C, as well as the influence of intellectual factors. Our research hypothesis is that the use of neural networks can help distinguish between ADHD-I and ADHD-C, and the inclusion of intelligence factors may increase the model prediction accuracy. The present study, conducted in Taiwan, collected data based on the SNAP-IV scale, the CPT-2 and CPT-3, and various intelligence tests. We used an artificial neural network to construct predictive models to distinguish between ADHD-I, ADHD-C, and the absence of ADHD.

## 2. Materials and methods

### 2.1. Participants and procedure

This was a retrospective study that analyzed medical records. The study was approved, and the waiver of informed consent was provided by the Taipei Medical University Joint Institutional Review Board (approval number: N202004034). The participants were patients who were assessed at any period between January 2011 and September 2020 in local hospitals in northern Taiwan.

1. *Inclusion Criteria:* This study included patients aged 6–17 years who received an ADHD-I or ADHD-C diagnosis. Children with ADHD are often diagnosed with other childhood and adolescent psychiatric disorders. According to a previous study, 66% ADHD patients had at least one comorbid psychiatric disorder, including learning disabilities, anxiety disorder, Tourette’s syndrome, etc. (Reale et al., 2017). This study tries to find the accuracy of artificial neural network for ADHD in the clinical situation. Therefore, in addition to the exclusion criteria, such as schizophrenia, organic psychosis, major depression disorder, if the ADHD patients have other comorbidity, they will still be included in the study. An individual may be assessed multiple times at different age. However, considering the illness severity, individual’s age, the teacher who finishing the SNAP-IV rating scales are all different at each evaluation, if this condition happens, the data will be included as another batch.
2. *Exclusion Criteria:* Patients were excluded if they were diagnosed with (i) neurological diseases, including disorders of the brain and central nervous system (e.g., epilepsy); (ii) intellectual disabilities (for patients who had taken an intelligence test); (iii) other serious psychological disorders, such as schizophrenia, bipolar disorder, and major depressive disorder; and (iv) physiological diseases potentially affecting attention and activity level.
3. *Diagnostic Procedure and Psychiatric Assessment Questionnaire:* Patients were first evaluated by psychiatrists from the Child and Adolescent Psychiatry Division to determine whether they had ADHD-I or ADHD-C, as defined by the DSM-V. In addition, the following psychiatric evaluations were conducted: (i) SNAP-IV scale evaluations by the patients’ teachers and parents to identify attention deficit and hyperactivity in patients; (ii) evaluations using the CPT-2 and the CPT-3; and (iii) intelligence tests, which were based on the Standard Progressive Matrices, the Colored Progressive Matrices, and the Wechsler Intelligence Scale.
4. *Grouping of Individuals:* According to the assessment results, individuals were divided into ADHD-I, ADHD-C, and control groups. The control group comprised patients who had received attention and activity level assessments in a hospital and for whom psychological assessment and psychiatric evaluation indicated no ADHD-I or ADHD-C.

**Figure 1:**
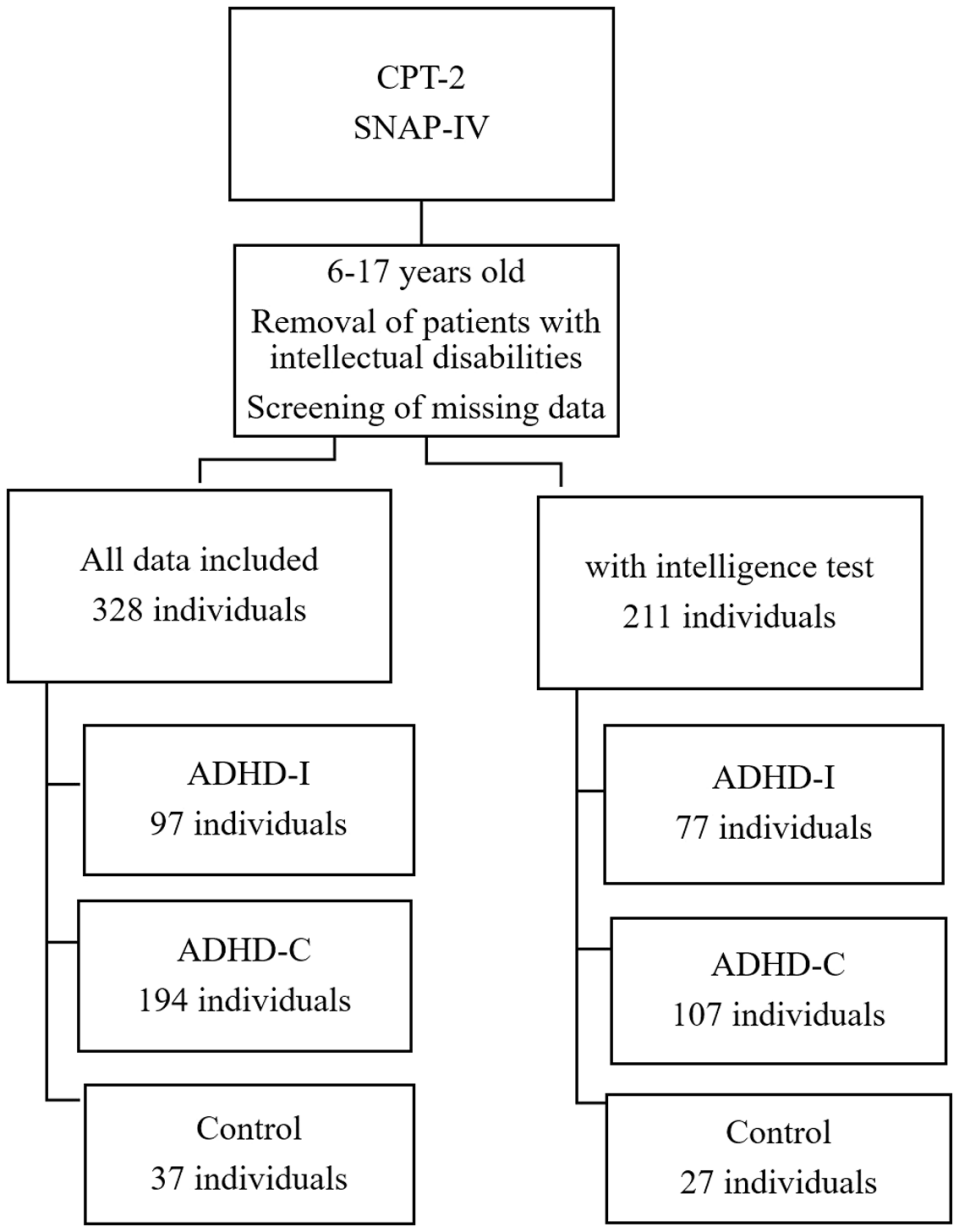
Grouping of CPT-2 individuals.

**Figure 2:**
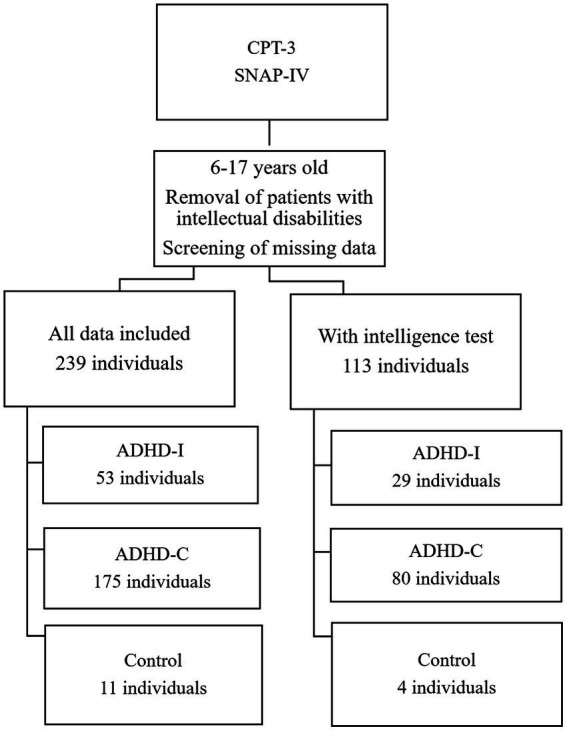
Grouping of CPT-3 individuals.

The data were categorized according to the two editions of the CPT, after erroneous or missing data points were removed. The process of grouping the individuals are presented in Figure 1 (for the CPT-2 group) and Figure 2 (for the CPT-3 group).

### 2.2. Selection of features

The following five items (30 indicators) were used in the modeling process:

1. Demographic items: sex and age; two indicators.
2. Intelligence test items: the overall score for all scales; one indicator.
3. SNAP-IV scale items: sum of scores for the inattention, hyperactivity, and oppositional subscales of the SNAP-IV scale evaluations by parents and teachers; six indicators.
4. CPT-2 items: omissions, commission, overall hit reaction time (hit RT), overall standard error (hit RT standard error), variability of standard error, detectability (d’), response style indicator (*β*), perseverations, hit reaction by block (hit RT block change), standard error by block (hit SE block change), reaction time by interstimulus interval (hit RT ISI change), and standard error by interstimulus interval (hit SE ISI change); 12 indicators.
5. CPT-3 items: detectability (d’), omissions, commission, perseverations, overall hit reaction time (hit RT), overall standard error (hit RT standard error), variability of standard error, hit reaction by block (hit RT block change), and reaction time by interstimulus interval (hit RT ISI change); nine indicators.

### 2.3. Building predictive models

This study used an artificial neural network. The data were segmented into training (70%), verification (15%), and testing (15%) data sets. To identify the optimal model, a neural network with perceptron in multiple layers was used to perform classification tasks. The hidden layer used the default setting with the default number of hidden neurons. Moreover, *k*-fold cross-validation was applied to ensure the consistency of machine learning results. Number of folds was selected as 5 for distinguishing ADHD-I and ADHD-C group in CPT-2 and CPT-3 set. Due to the data with the intelligence test and the control group is relatively small, number of folds was selected as 3, and also for distinguishing ADHD-I, ADHD-C and Control group in CPT-2 set.

### 2.4. Model evaluation and confusion matrix

After the neural network was trained on the data, the confusion matrix was used to evaluate model performance (Table 1). By using the model, the neural network will predict ADHD-C or ADHD-I (predictive classification) with input factors and compare the actual diagnosis (actual classification). If the prediction is consistent with the actual diagnosis, it is true positive or true negative. If the prediction is inconsistent, it is false positive or false negative. After that, we calculate recall, precision, and overall accuracy.

1. Recall: The case was actually positive and then predicted to be positive. The ratio is TP/(TP + FN).
2. Precision: The case was predicted to be positive and then actually positive. The ratio is TP/(TP + FP).
3. Accuracy: The ratio is TP + TN/(TP + TN + FP + FN).

**Table 1:** Confusion matrix.

		Actual classification		Total
		Positive	Negative	
Predictive classification	Positive	True positive (TP)	False positive (FP)	TP + FP
	Negative	False negative (FN)	True negative (TN)	FN + TN
Total		TP + FN	FP + TN	TP + FN + FP + TN

## 3. Results

This study included 328 participants with CPT-2 and 239 participants with CPT-3. Participants were divided into ADHD-I, ADHD-C, and control groups according to the assessment results.

Table 2 shows the sociodemographic and clinical characteristics of all individuals. The age of control croup at evaluation is significant older then ADHD group. In the CPT-2 set, the intelligence of ADHD-I and control group is lower than ADHD-C group. In the CPT-3 set, the intelligence of control group is lower than ADHD-C group, but nonsignificant. This may be due to the control group is very small (*n* = 4).

**Table 2:** Sociodemographic and clinical characteristics of individuals.

	CPT-2 set (*n* = 328)				CPT-3 set (*n* = 239)			
	ADHD-I	ADHD-C	Control	*p*	ADHD-I	ADHD-C	Control	*p*
	(*n* = 97)	(*n* = 194)	(*n* = 37)		(*n* = 53)	(*n* = 175)	(*n* = 11)	
Sex, *n* (%)								
Male	60 (61.86%)	163 (84.02%)	30 (81.08%)		37 (69.81%)	151 (86.29%)	9 (81.82%)	
Female	37 (38.14%)	31 (15.98%)	7 (18.92%)		16 (30.19%)	24 (13.71%)	2 (18.18%)	
Age (years), *M* (SD)	8.79 (2.28)	8.26 (2.12)	9.68 (3.27)^a^	0.0018^**^	9.87 (2.92)^a^	8.87 (2.38)	11 (2.56)^a^	0.0023^**^
Intelligence, *M* (SD)	90.86 (13.11)^a^	97.42 (13.86)	88.93 (13.65)^a^	0.0008^***^	90.28 (11.34)	92.24 (11.90)	77.75 (9.60)	0.0516
	(*n* = 77)	(*n* = 107)	(*n* = 27)		(*n* = 29)	(*n* = 80)	(*n* = 4)	
SNAP-IV—Parent								
Inattention score, *M* (SD)	15.02 (5.29)^b^	16.32 (5.14)^b^	10.19 (3.77)	<0.0001	16.43 (4.52)^b^	16.68 (5.24)^b^	10.63 (5.22)	0.0008^***^
Hyperactivity/impulsivity score, *M* (SD)	5.98 (3.96)^c^	13.82 (5.61)^b^	5.70 (3.81)	<0.0001	6.68 (5.25)^c^	14.11 (5.66)^b^	3.91 (3.75)	<0.0001
Oppositional score, *M* (SD)	6.58 (5.59)^c^	11.38 (5.42)^b^	7.05 (5.52)	<0.0001	7.15 (5.82)^c^	11.33 (5.91)^b^	4.91 (5.65)	<0.0001
SNAP-IV—Teacher								
Inattention score, *M* (SD)	15.69 (6.09)^b^	16.28 (6.14)^b^	8.40 (4.43)	<0.0001	15.36 (6.04)^b^	17.06 (5.83)^b^	8.45 (3.21)	<0.0001
Hyperactivity/impulsivity score, *M* (SD)	5.10 (4.39)^c^	14.01 (7.53)^b^	3.22 (2.89)	<0.0001	4.60 (6.12)^c^	13.37 (6.97)^b^	2.36 (3.41)	<0.0001
Oppositional score, *M* (SD)	3.40 (3.90)^c^	9.96 (6.87)^b^	3.7 (4.20)	<0.0001	3.79 (4.95)^c^	8.77 (6.07)^b^	1.72 (2.90)	<0.0001

*p < 0.05, **p < 0.01, ***p < 0.001. ^a^Indicates significant difference between ADHD-I or Control group and ADHD-C group in Scheffe post hoc test. ^b^Indicates significant difference between ADHD-I or ADHD-C group and Control group in Scheffe post hoc test. ^c^Indicates significant difference between ADHD-I group and ADHD-C group in Scheffe post hoc test.

Table 3 presents the overall accuracy of the models at different eigenvalues. In models M1–M4, data were used to distinguish between ADHD-I and ADHD-C. The combination of demographic information, SNAP-IV scale results, and CPT-3 results yielded higher accuracy (overall accuracies of 90.46 and 89.44% for the training and testing sets, respectively) relative to the combination of demographic information, SNAP-IV scale results, and CPT-2 results (overall accuracies of 88.75 and 85.56% for the training and testing sets, respectively). The addition of intelligence data into the model improved the overall accuracy (91.03% in the combination with CPT-2, 93.57% in the combination with CPT-3) for the training set, but not for the testing set (84.77 and 89.00% in the models accounting for CPT-2 and CPT-3, respectively).

**Table 3:** The overall accuracy of models by using different indicators.

Model*	Demographic information	Intelligence test	SNAP-IV scale	CPT-2	CPT-3	Number of indicators	Overall accuracy	
							Training set	Testing set
M1	ˇ		ˇ	ˇ		20	88.75%	85.56%
M2	ˇ	ˇ	ˇ	ˇ		21	91.03%	84.77%
M3	ˇ		ˇ		ˇ	17	90.46%	89.44%
M4	ˇ	ˇ	ˇ		ˇ	18	93.57%	89.00%
M5	ˇ		ˇ	ˇ		20	86.74%	77.43%
M6	ˇ	ˇ	ˇ	ˇ		21	89.08%	74.87%

^*^M1–M4 aimed to distinguish ADHD-I and ADHD-C. M5–M6 aimed to distinguish between the ADHD-I, ADHD-C, and control groups.

We also used CPT-2 data to distinguish between participants in the ADHD-I, ADHD-C, and control groups. In the M5 model, the overall accuracy was 86.74% for the training set and 77.43% for the testing set. In the M6 model, which included intelligence, the overall accuracy was 89.08% for the training set and 74.87% for the testing set.

Table 4 presents the mean recall, precision, and accuracy of the cross-validated training set and testing set using different models. The M1 model, which included demographic information, SNAP-IV scale results, and CPT-2 results yielded an ADHD-I recall ratio of 85.31% and an ADHD-C recall ratio of 90.47% for the training set. By adding intelligence into the M2 model, the ADHD-I recall ratio was 89.59% and the ADHD-C recall ratio was 92.06% for the training set. Moreover, the ADHD-I recall ratio was 80.42% and the ADHD-C recall ratio was 88.18% for the testing set.

**Table 4:** Model performance of the cross-validated training set and testing set.

Model^*^	Training set					Testing set				
	ADHD-I	ADHD-I	ADHD-C	ADHD-C	Overall accuracy	ADHD-I	ADHD-I	ADHD-C	ADHD-C	Overall accuracy
	Recall	Precision	Recall	Precision		Recall	Precision	Recall	Precision	
M1 (*n* = 291)	85.31%	81.75%	90.47%	92.49%	88.75%	80.42%	77.67%	88.18%	90.05%	85.56%
M2 (*n* = 184)	89.59%	89.11%	92.06%	92.51%	91.03%	83.08%	81.10%	85.98%	87.70%	84.77%
M3 (*n* = 228)	70.72%	85.80%	96.43%	91.63%	90.46%	65.62%	84.97%	95.98%	90.48%	89.44%
M4 (*n* = 109)	84.27%	90.69%	96.83%	94.53%	93.57%	72.23%	86.15%	95.03%	90.53%	89.00%

^*^The CPT-3 control groups were too small (*n* = 11 and 4 in models 3 and 4, respectively) for the confusion matrix to be analyzed. We only selected participants in the ADHD-I and ADHD-C groups to analyze the confusion matrix.

Table 4 also presents the M3 model, which included demographic information, SNAP-IV scale results, and CPT-3 results; in this model, the ADHD-I recall ratio was 70.72% and the ADHD-C recall ratio was 96.43% for the training set. Adding intelligence into the M4 model yielded an ADHD-I recall ratio of 84.27% and an ADHD-C recall ratio of 96.83% for the training set. Moreover, the ADHD-I recall ratio was 72.23% and the ADHD-C recall ratio was 95.03% for the testing set. In the training set, the overall accuracy of the M2 and M4 models in detecting ADHD-I increased compared with the overall accuracy of the M1 and M3 models in detecting ADHD-I, but the overall accuracy of the M2 and M4 models in the testing set was not different from that of the M1 and M3 models.

Tables 5, 6 presents mean recall, precision, and accuracy of cross-validation between the training set and the testing set using the CPT-2 data to distinguish between the ADHD-I, ADHD-C, and control groups. The M5 model, which included demographic information, SNAP-IV scale results, and CPT-2 results, yielded an ADHD-I recall ratio of 82.00%, an ADHD-C recall ratio of 90.90%, and a control recall ratio of 77.00% for the training set. For the testing set, the ADHD-I recall ratio was 67.05%, the ADHD-C recall ratio was 85.09%, and the control ratio was 65.17%.

**Table 5:** Model performance of the cross-validated training set with CPT-2.

Model	Training set						
	ADHD-I	ADHD-I	ADHD-C	ADHD-C	Control	Control	Overall accuracy
	Recall	Precision	Recall	Precision	Recall	Precision	
M5 (*n* = 328)	82.00%	80.26%	90.90%	91.23%	77.00%	80.28%	86.74%
M6 (*n* = 211)	86.99%	86.38%	92.97%	93.39%	79.63%	81.22%	89.08%

**Table 6:** Model performance of the cross-validated testing set with CPT-2.

Model	Testing set						
	ADHD-I	ADHD-I	ADHD-C	ADHD-C	Control	Control	Overall accuracy
	Recall	Precision	Recall	Precision	Recall	Precision	
M5 (*n* = 328)	67.05%	69.05%	85.09%	86.11%	65.17%	58.42%	77.43%
M6 (*n* = 211)	63.64%	70.05%	85.00%	83.47%	66.67%	56.96%	74.87%

Adding intelligence into the M6 model yielded an ADHD-I recall ratio of 86.99%, an ADHD-C recall ratio of 92.97%, and a control ratio of 79.63% for the training set. For the testing set, the ADHD-I recall ratio was 63.64%, the ADHD-C recall ratio was 85.00%, and the control ratio was 66.67%.

## 4. Discussion

This study observed that the combination of demographic information, SNAP-IV scale results, and CPT-2 results yielded an overall accuracy of 88.75% in the training set and 85.56% in the testing set. By contrast, the combination of demographic information, the SNAP-IV scale, and the CPT-3 results yielded an overall accuracy of 90.46% in the training set and 89.44% in the testing set. The use of the CPT-3 resulted in higher model accuracy for distinguishing between ADHD-I and ADHD-C. However, because the data used in this study were collected between 2011 and 2020, the training results may have been affected by the relatively poor consistency of earlier data. This explains why, despite the comparable overall accuracy of the two training sets (both 88–90%), the accuracy of the CPT-2 testing set decreased.

The addition of the results from the intelligence tests into the model increased ADHD-I recall (CPT-2: from 85.31 to 85.59%, CPT-3: from 70.75 to 84.27%; CPT-2 for distinguishing between the three groups: from 82.00 to 86.99%) and slightly increased the overall accuracy in both training sets. However, in the testing set, the change in ADHD-I recall was inconsistent and produced no significant increase in the overall accuracy.

Previous studies showed if adults with ADHD and higher IQ (>110) performed significantly better on CPT than those with ADHD and standard IQ (Milioni et al., 2017; Baggio et al., 2020). In children, a study showed ADHD children, aged 5–15 years, with higher IQ (>120), performed superiorly to the standard IQ ADHD children, with regard to omission and commission errors on the visual–auditory CPT (Park et al., 2011). However, a study found that even ADHD children with higher IQ, still performed worse on the executive function tests (Stroop color-word and trail-making tests) than normal control group with a high IQ (He et al., 2013). Relative to control participants, children with ADHD and a high IQ still were rated by parents as having more functional impairments across a number of domains (Antshel et al., 2007). When patients were transferred to hospital for evaluation, they may had more sever ADHD symptoms observed by the parents or teachers with higher behavior rating scales, regardless of whether children with higher IQ or not. Therefore, intelligence may be not a strong factor using for machine learning to distinguish ADHD. Another possible explanation is that the inclusion of results from the intelligence tests reduced the sample size, which in turn caused overfitting. Overall, it is difficult to judge whether intelligence predicts ADHD-I in the absence of more data than what were available in this study.

The patients in this retrospective study were brought to the hospital for assessment after the patient’s parents and teachers observed difficulties with their concentration, activity level, and impulse control. Therefore, the control group was relatively small, and the patients may differ from their counterparts in the general population. They were referred to hospital for evaluation at older age, and the intelligence of the control group is lower than the ADHD group. This constitutes a limitation of the present study. The overall accuracies for CPT-2 were 86.74 and 77.4% in the training and testing sets, respectively. Although the overall accuracy among participants with CPT-2 was acceptable, future studies should include more patients to construct a more stable model.

ADHD may be caused by various factors, such as those pertaining to the environment and one’s genetic makeup and personal characteristics, and ADHD is prone to different clinical manifestations between patients because of the inherent uniqueness in the relationship between an individual and their environment. One study assessed brainwave examinations and reported positive and negative predictive powers of 98 and 76%, respectively. This indicates that a very high proportion of brainwave abnormalities are observed in ADHD patients (Monastra et al., 2001). However, even if the brainwaves are normal, 24% of individuals may still have ADHD. Furthermore, data obtained from the current state of brainwave-measurement technology cannot be used to correctly distinguish between ADHD-I and ADHD-C. At present, the diagnosis of ADHD-C and ADHD-I by psychiatrists is based not only on symptoms but also on changes in the patient’s academic performance and social functioning and on the negative effects of symptoms. One study applied the LightGBM algorithm in machine learning with Conners’ Adult ADHD Rating Scales (26 items) to differentiate subjects with ADHD, obesity, problematic gambling, and a control group with a global accuracy of 0.80; precision ranged between 0.78 (gambling) and 0.92 (obesity), recall between 0.58 for obesity and 0.87 for ADHD. The combination of behavior scales, psychological tests and machine learning may offer benefit for variable diagnosis in clinical (Christiansen et al., 2020).

Because this was a retrospective study of patients who presented to the hospital for assessment, patients in the control group were few in number and may differ from the average person in the general population. At the point of evaluation, patients may have other comorbidities, and some patients may have received medication which may also affect the results of the CPT test and the teacher’s and parent’s SNAP-IV scales. In a previous study, it was shown that family education, household income, and social deprivation index may intelligence-independently increase the risk of an ADHD diagnosis. These factors were not available in our study and may need to be explored in the future (Michaëlsson et al., 2022). Moreover, this study was based on hospital data from 2011 to 2020, and the situations of other hospitals and communities are still unclear. In addition, the data points in this study were manually entered one by one, and some data may still be missing or incorrect. Finally, the diagnosis of ADHD may be inconsistent between clinicians, which could affect the accuracy of the data and the process of machine learning. These limitations should be addressed and resolved in future studies.

## 5. Conclusion

Overall, artificial neural networks can be used to integrate complicated clinical data, including those on age, sex, intelligence, SNAP-IV scale results (obtained through parent and teacher observations), and computer-based test results. This study’s method had a 74–89% accuracy in distinguishing between the ADHD-I, ADHD-C, and control groups and an 85–90% accuracy (which is sufficient for real-world applications) in distinguishing between the ADHD-I and ADHD-C groups. Therefore, artificial intelligence, machine learning, and deep learning are expected to be useful for ADHD diagnosis in the future. If future studies can obtain a large, accurate clinical data set for machine learning, the accuracy can be improved. The machine learning model can help physicians distinguish between patients with ADHD-I, with ADHD-C, and without ADHD more quickly, making treatment and identification more timely.

## Data availability statement

The raw data supporting the conclusions of this article will be made available by the authors, without undue reservation.

## Ethics statement

The studies involving human participants were reviewed and approved by Taipei Medical University Joint Institutional Review Board. Written informed consent from the participants’ legal guardian/next of kin was not required to participate in this study in accordance with the national legislation and the institutional requirements.

## Author contributions

I-CL designed the idea for the manuscript, analyzed the data, and wrote the manuscript. TK and H-WC designed and coordinated the study. I-CL, S-CC, and Y-JH collected and managed the data. H-WC revised the manuscript. All authors contributed to the article and approved the submitted version.

## Funding

The study was supported by the Ministry of Science and Technology, Taiwan, R.O.C., under grant numbers MOST109-2221-E-038-011 and MOST110-2221-E-038-006.

## Conflict of interest

The authors declare that the research was conducted in the absence of any commercial or financial relationships that could be construed as a potential conflict of interest.

## Publisher’s note

All claims expressed in this article are solely those of the authors and do not necessarily represent those of their affiliated organizations, or those of the publisher, the editors and the reviewers. Any product that may be evaluated in this article, or claim that may be made by its manufacturer, is not guaranteed or endorsed by the publisher.
